# A Public Duty to Practitioners

**Published:** 1919-03-22

**Authors:** 


### A PUBLIC DUTY TO PRACTITIONERS.

The recently published letters signed by the respective Presidents of the Royal College of Physicians and the Royal College of Surgeons in both London and Edinburgh put the essential points with admirable clearness, and it is hoped that their words will meet with the appreciation and understanding of every home in England.

The accepted principle throughout the country has been to give returning soldiers and sailors the opportunity of reinstatement in positions they voluntarily resigned to play their part in the world-struggle, and though this may be done by the co-operation of a limited number of people, namely, the employers, yet in a profession so essentially personal as medicine, the same direct methods can never apply. Even if the early recommendations of leading medical bodies have been carried out, and remaining practitioners have with full loyalty endeavoured to maintain the practices of those who responded to the call, there are a number of factors which, in so long an absence as four-and-a-half years, must inevitably have reduced their extent to a considerably lower dimension than in 1914. It is a well-known fact that many are finding themselves in the greatest difficulties, threatened with serious financial embarrassment and, in practically every instance, through no fault of their own.

With regard to salaried appointments one has every confidence that governing bodies will act strictly upon the reinstatement principle, and one hopes that the delay observed in some instances arises from causes other than indecision. It is to the people, the patients themselves, that it is so necessary for the facts to be demonstrated. Justice must be done to each returning man, and, in this instance, the public has its accomplishment largely in its own hands. In no section of the community was response to the call of war more ready, and the medical work compares with the finest produced throughout the past years' trials. People should remember the doctor to whom they gave their trust in 1914, and all that he has, though absent, done for them in the meantime. Before the war they accepted his guidance. His experience is now many times increased by days of concentrated, cruel learning. Surely it is the honourable duty of every one to seek, whenever circumstance and loyalty permit, the care and advice of their pre-war doctor.

